# The complete mitochondrial genome of golden yellow snakehead fish, *Channa argus*

**DOI:** 10.1080/23802359.2018.1522981

**Published:** 2018-10-05

**Authors:** Shu-Ren Zhu, Qing-Lei Meng, Yong-An Zhu, Li An, Long-Gang Zhang, Zhen-Hai Yu, Pei-Sheng Fu, Yuan-Yuan Zhang, Zhi-Shan Zhang

**Affiliations:** Shandong Freshwater Fisheries Research Institute, Jinan, China

**Keywords:** Channa argus, golden yellow color, complete mitochondrial genome

## Abstract

We sequenced and characterized the complete mitochondrial genome of golden yellow snakehead fish, *Channa argus.* The mitogenomes contained the typical complement of 13 protein-coding genes, 22 transfer RNAs (tRNAs), 2 ribosomal RNAs (rRNAs), and a non-coding control region. They share the same gene arrangement pattern that was identical with most vertebrates. The entire mitochondrial DNA molecule of golden yellow snakehead fish was 16,558 bp long. All information reported in this article will be a useful source of sequence information for general molecular and evolutionary studies of the family Channidae.

*Channa argus*, commonly called the snakehead fish, belongs to the family Channidae. In many areas of the world, the snakehead fish is a freshwater cultured species which is famous for its fast growth, nutrition, and economic value, especially in China. Its normal body color is grayish black, however, we have bred golden yellow individuals during the breeding process in Ju County. Here we reported the entire mtDNA sequences of golden yellow snakehead fish in order to provide genetic tools for the conservation and breeding of golden yellow snakehead fish.

In this study, the complete mitochondrial genome of the golden yellow snakehead fish was amplified and sequenced. The fish sample was collected from Ju County, China (35°35'42" latitude, 118°48'36″longitude) and is deposited in Shandong Provincial Key Laboratory of Freshwater Genetics and Breeding (36°41′10″ latitude,116°51′3″ longitude) with identifier CA-G-01. The complete mitochondrial genome was amplified using 22 primer pairs, which were designed using Primer Premier 5.0 based on *C. argus* (GenBank accession number JX978723). And PCR products were sequenced by Map Biotechnology Co., Ltd. The mitogenome of the golden yellow snakehead fish was 16,558 bp long. All newly determined sequences from the present study were deposited in the GenBank database (MG751766).

The structural organization and location of different feature in these mt genomes conformed to the common vertebrate mt genome model and consisted of 13 protein-coding genes, 2 rRNAs, 22 tRNAs, and 1 putative control region (Liu and Cui 2009). Like other vertebrates, most of the genes of the golden yellow snakehead fish were encoded on the H-strand, with only ND6 and eight tRNAs (*Gln, Ala, Asn, Cys, Tyr, Ser, Glu* and *Pro*) located on the L-strand, and all the genes were similar in length to those in other bony fishes (Miya et al. 2003). The gene order was identical to that obtained for other vertebrates. Nucleotide base composition of the complete sequences was the following: 27.23% for A, 31.60% for C, 16.98% for G, 24.20% for T in the golden yellow snakehead fish. Except for COX1 with a GTG start codon, the remaining 12 PCGs start with an ATG codon.

Phylogenetic analysis was performed by MEGA 6.06 (Tamura et al. 2013) based on the concatenated nucleotide sequences of 12 protein-coding genes (except ND6) from the mitogenomes of the snakehead fish and those of 10 closely related species belonging to three families Channidae, Percichthyidae, and Lutjanidae. The maximum likelihood tree (Figure 1) showed that *C. argus* first clustered together with *C. maculata, C. marulius, C. asiatica, C. striata,* and *C. lucius,* and formed the family *Channidae*, and then they constituted a sister-group relationship with the other two families.

**Figure 1.**
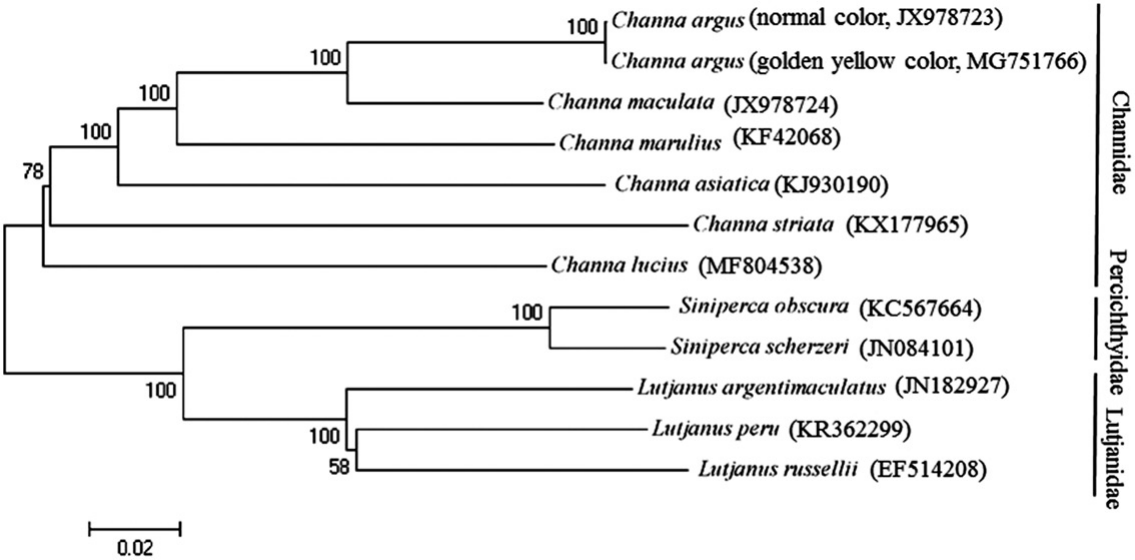
Maximum likelihood (ML) phylogenetic trees inferred from the nucleotide sequence data of mitogenomic 12 protein-coding genes (except ND6).
